# Dye-Perfused Human Placenta for Simulation in a Microsurgery Laboratory for Plastic Surgeons

**DOI:** 10.1055/a-2113-4182

**Published:** 2023-11-01

**Authors:** Laura C. Zambrano-Jerez, Karen D. Díaz-Santamaría, María A. Rodríguez-Santos, Diego F. Alarcón-Ariza, Genny L. Meléndez-Flórez, Mónica A. Ramírez-Blanco

**Affiliations:** 1 Division of Plastic and Reconstructive Surgery, Universidad Industrial de Santander, Hospital Universitario de Santander, Santander, Colombia; 2 Division of Plastic and Reconstructive Surgery, Universidad Industrial de Santander, Hospital Internacional de Colombia, Santander, Colombia

**Keywords:** microsurgery, plastic surgery, simulated training, placenta, anatomical models

## Abstract

In recent decades, a number of simulation models for microsurgical training have been published. The human placenta has received extensive validation in microneurosurgery and is a useful instrument to facilitate learning in microvascular repair techniques as an alternative to using live animals. This study uses a straightforward, step-by-step procedure for instructing the creation of simulators with dynamic flow to characterize the placental vascular tree and assess its relevance for plastic surgery departments. Measurements of the placental vasculature and morphological characterization of 18 placentas were made. After the model was used in a basic microsurgery training laboratory session, a survey was given to nine plastic surgery residents, two microsurgeons, and one hand surgeon. In all divisions, venous diameters were larger than arterial diameters, with minimum diameters of 0.8 and 0.6 mm, respectively. The majority of the participants considered that the model faithfully reproduces a real microsurgical scenario; the consistency of the vessels and their dissection are similar in in vivo tissue. Furthermore, all the participants considered that this model could improve their surgical technique and would propose it for microsurgical training. As some of the model's disadvantages, an abundantly thick adventitia, a thin tunica media, and higher adherence to the underlying tissue were identified. The color-perfused placenta is an excellent tool for microsurgical training in plastic surgery. It can faithfully reproduce a microsurgical scenario, offering an abundance of vasculature with varying sizes similar to tissue in vivo, enhancing technical proficiency, and lowering patient error.

## Introduction

A new age in reconstructive surgery has begun, thanks to microsurgery, which has emerged as a very relevant specialty in the surgical field. A variety of simulation models, including synthetic, ex vivo, and in vivo models, have been reported in recent decades for microsurgical training, particularly those that are focused on the microvascular area. 1 Ex vivo models include cadaveric human specimens, bovine or human placentas, chicken or turkey wings, and thighs. Rats are typically used for in vivo studies. 1 2 3 One of its limitations is that the use of live animals in microsurgery laboratories is restricted by law, requires more infrastructure and qualified staff, raises expenses, and has serious ethical implications. 2 3 4

Currently, the use of the human placenta has been described as part of comprehensive microsurgical training programs that combine the use of multiple synthetic, ex vivo, and in vivo models in order to promote progressive learning and limit the use of live animals. 4

**Fig. 1:**
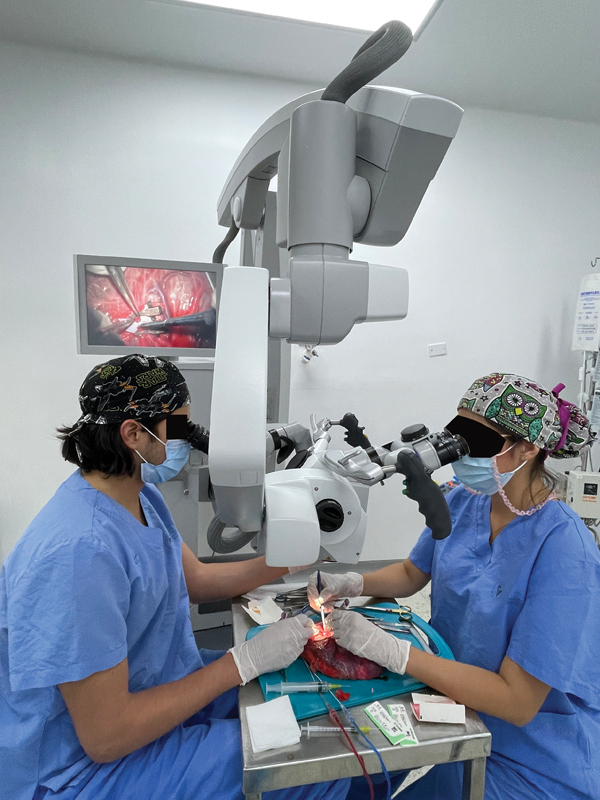
Microsurgery laboratory for plastic surgery residents.

The present study describes a technique for preparing the human placenta with color perfusion for use as a simulation model in the basic microsurgery laboratory ( Fig. 1 ) and presents the results obtained from the evaluation carried out by residents of a plastic surgery program to determine the reliability of the model. At the same time, it presents a characterization of the placental vascular tree that allows for recognition of its applicability in microvascular repair training.

## Methods

This study was carried out with the approval of the Research Ethics Committees. After applying informed consent to patients from the obstetrics service, 18 nonpathological fresh human placentas were obtained with preserved morphology of the allantois and the residual segment of the umbilical cord; all obstetric patients from whom placentas were obtained had negative results on screening for common infections.

After obtaining the fresh placentas we took the measurement of the central placental diameter and thickness. Additionally, the location of the umbilical cord was determined.

### Preparation of the Human Placenta as a Simulation Model

For the preparation technique, we used fresh placentas in the first hour after delivery and divided the process into two steps: the first that includes the cleaning of the placenta after delivery and the consequent removal of residual clots; and the second that consists of the perfusion of dyes through the vascular tree.

#### Cleaning and Clot Removal

After the delivery period, the placentas were collected and irrigated 7 cm from the surface with water at 36°C, the umbilical cord was sectioned, and the chorioamniotic membrane was subsequently removed to improve visualization of the blood vessels.

The umbilical vein and arteries were identified and these vessels were cannulated with 3.5- and 2.5-Fr umbilical catheters, respectively; the catheter was attached to a container of saline solution. In order to clean the blood vessels and remove blood clots, a first wash of the lumen with saline solution was performed until a translucent coloration of the blood vessels was achieved, then a second irrigation was performed with a dilution of 5000 IU of sodium heparin in 500 mL of saline solution. The catheters were attached to the umbilical cord using 0–0 silk.

**Fig. 2:**
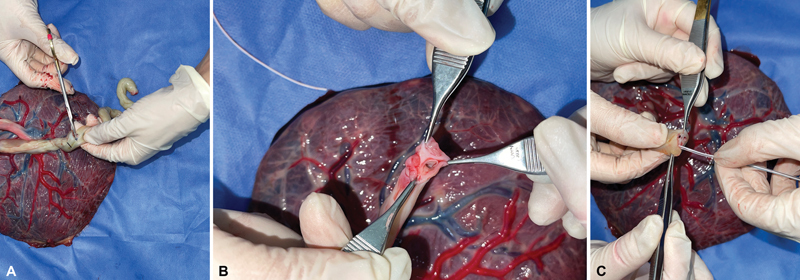
Preparation of the human placenta as a simulation model. ( **A** ) Axial section of the umbilical cord. ( **B** ) Identification of the umbilical arteries and umbilical vein. ( **C** ) Umbilical vein catheterization.

The approximate duration of the preparation phase was 15 minutes ( Fig. 2 ).

#### Dye Instillation

**Fig. 3:**
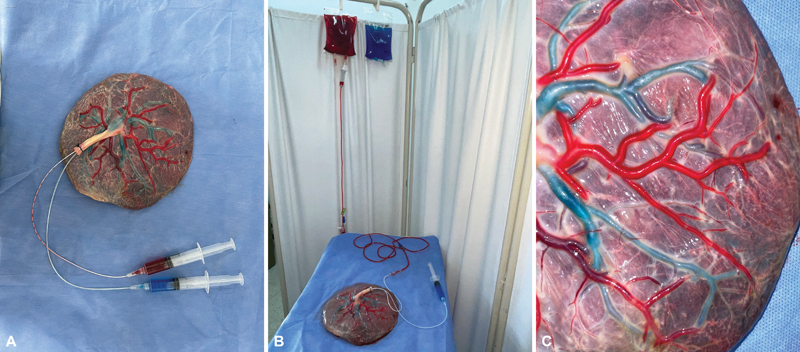
Dyes Perfusion in the placental model. ( **A** ) Fixation of the perfusion system. ( **B** ) Infusion of the dye dilution in saline through the perfusion system. ( **C** ) Dye placental vessels.

In the absence of visible clots, carmine red (food colorant easily available) and methylene blue dye dilutions were instilled into the arterial and venous systems, respectively. For carmine red, a dilution of 1 mL in 500 mL of saline was used, and for methylene blue, a dilution of 2 mL in 500 mL of saline was used. This infusion was carried out continuously until the entire vascular tree was pigmented. The approximate duration of this phase of the preparation was 1 hour ( Fig. 3 ).

After finished the preparation process, while the microsurgery sessions started, the tissue was stored under a wet laparotomy towel with saline solutions for 1 hour at 20 to 22°C, then the training session was performed; at the end, the model was discarded. In this laboratory we use used fresh preparations for each sessions, but it is possible stored at a temperature 4 to 10°C for 6 to 24 hours 5 6 or 3°C and use during 4 days or 1 week, 7 or frozen at −18° C and increase the viable time for up to 40 days. 1 6 During the practice, it was found that the placenta can be use at 20 to 22°C without refrigeration at least for 6 to 8 hours before the tissue turns friable and delicate. Regarding the preparation process, most of the previous studies describe the use of saline solution 8 and heparine 6 and then perfusing it with colored normal saline (with different types of dye solutions). 1 3 7 Other reports have used silicone plus catalyst for simulation of tumors. 9

### Measurement of the Placental Vascular Tree

**Fig. 4:**
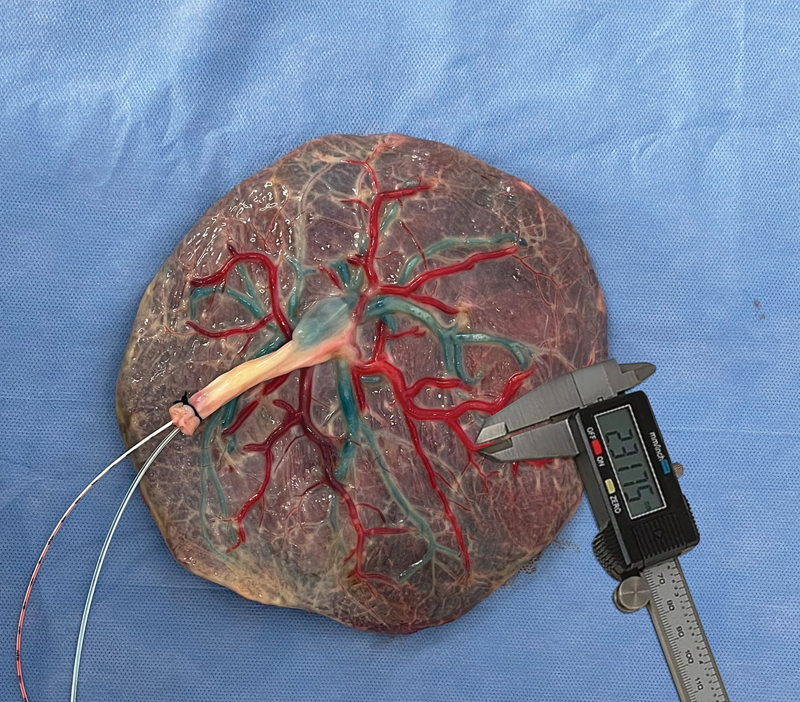
Measurement of the diameter of the placental vessels using the digital pachymeter.

Taking as a reference the classification of the placental vascular tree proposed by Bekov 10 11 and adopted by other authors, the diameter of the arterial and venous vessels of the placental surface was measured using a digital pachymeter prior to perfusion with saline solution at 80 mm Hg of pressure controlled by a sphygmomanometer, 12 obtaining measurements up to four arterial and venous divisions ( Fig. 4 ). The results are presented in Table 1 .

**Table 1:** Diameters of the placental vessels by division

Division		Mean (mm)	SD (mm)	Minimum (mm)	Maximum (mm)
Arteries	A1	5.83	± 0.87	4.60	8.00
	A2	4.09	± 0.98	2.30	6.50
	A3	2.78	± 0.80	1.80	4.30
	A4	1.38	± 0.59	0.60	2.60
Veins	V1	8.07	± 0.86	7.00	10.00
	V2	5.97	± 1.14	4.00	8.60
	V3	3.77	± 1.10	2.20	6.00
	V4	2.03	± 0.59	0.80	3.00

Abbreviation: SD, standard deviation.

Note: 1, first division; 2, second division; 3, third division; 4, fourth division.

### Evaluation of the Reliability of the Placental Model

In a protocol for vascular microneurosurgery training Dr. Del Maestro et al 1 find human placenta indicated as an accurate and reproducible model. They designed a survey with five consecutive questions and concluded the validity of this model across different specialties, regardless of the experience of the trainees. We applied the survey, and at the end, two open questions were asked to expand the information about disadvantages and difficulties of the model. Twelve participants took the survey: nine plastic surgery residents in their first up to fourth year of residency; and three faculty: two microsurgeons and one hand surgeon.

Participants were divided into three categories according to their expertise in microsurgery: novice (those in their first and second year as residents), intermediate (those that were in their third and fourth year as residents), and advanced (specialists with experience in microsurgery). The questionnaire consisted of seven questions, five of which used a Likert-type scale, and two open-ended questions. The questions were intended to evaluate the validity and reliability of the model for training in microsurgery 1 ( Table 1 ).

### Statistical Analysis

A univariate analysis was carried out using measures of tendency to center and dispersion. Quantitative variables were described by mean and confidence interval (normality was evaluated using a Shapiro–Wilk test); qualitative variables were described by their absolute and relative frequencies and their confidence interval.

A bivariate analysis was performed comparing the evaluated characteristics of the placenta with the experience of the evaluator using the chi square test, which is a statistical test used to compare observed and expected results, to identify whether a disparity between actual and predicted data are due to chance or to a link between the variables under consideration.

## Results

### Characterization of the Placental Vascular Tree

The 18 placentas that were obtained had an average weight of 541.39 ± 73.90 g (maximum 715–minimum 420 g). The mean diameter of the placental surface was 18.83 ± 1.65 cm and the mean thickness evaluated in the central portion of the placenta was 1.55 ± 0.31 cm. A total of 56% of the obtained placentas presented an eccentric position of the umbilical cord; the rest corresponded to the centric position.

The mean arterial diameter of the first division was 5.83 ± 0.87 mL, in the second division it was 4.09 ± 0.98 mL, in the third division it was 2.78 ± 0.80 mL, and finally, in the fourth division it was 1.38 ± 0.59 mL. The largest arterial diameter in all divisions was 8 mL, and it was documented in the first division artery. The smallest arterial diameter in all divisions was 0.6 mL, located in a fourth division artery ( Table 1 ).

In terms of venous vessels, the mean venous diameter in the first division was 8.07 ± 0.86 mL, in the second division it was 5.97 ± 1.14 mL, in the third division it was 3.77 ± 1.10 mL, and in the fourth division it was 2.03 ± 0.59 mL. The largest venous diameter was higher compared with the largest arterial diameter, being 10 mL and located in the first venous division. In the same way, the smallest venous diameter was 0.8 mL, which was higher than the smallest recorded arterial diameter, which was located in the fourth venous division ( Table 1 ).

### Reliability of the Placenta as a Simulation Model

**Graph 1:**
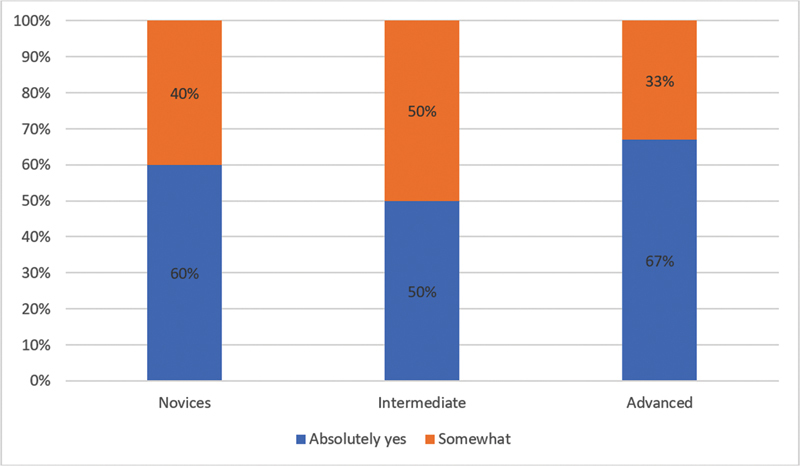
Question number 1: Results by subgroups. Do you think that the proposed training model faithfully reproduces a possible real microsurgical scenario?

**Graph 2.**
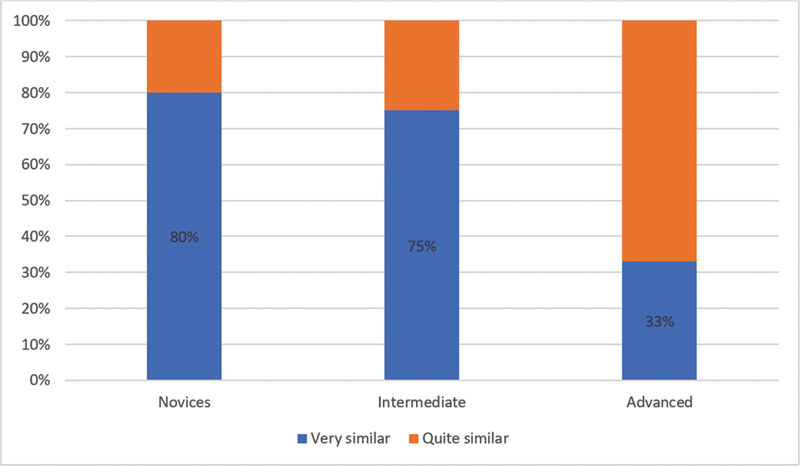
Question number 2: Results by subgroups. According to your surgical experience, is the consistency of the placental vessels comparable to the one of the in vivo tissues?

**Graph 3.**
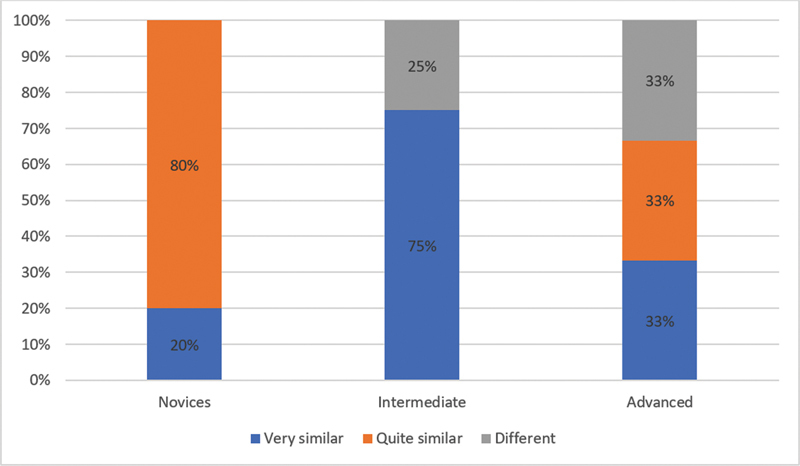
Question number 3: Results by subgroups. Do you think that the dissection of human placenta vessels is similar to in vivo vessels dissection?

Twelve participants took the survey: nine plastic surgery residents in their first up to fourth year of residency and three faculty: two microsurgeons and one hand surgeon. The general results can be seen in Table 2 , all the participants took the survey ( *n* = 12). A total of 58% ( *n* = 7) of the participants were men and the mean age was 32 years old. Of the total number of participants, 58% ( *n* = 7) responded absolutely yes and 42% ( *n* = 5) somewhat with question number 1, which inquired about the model's ability to perform a faithful reproduction of a real microsurgical scenario; in the novice group, 60% of participants absolutely yes; in the intermediate group, 50% somewhat agreed; and in the more experienced group, 67% somewhat agreed ( Graph 1 ). In question number 2, 42% ( *n* = 5) of the participants answered that the consistency of the placental vessels were very similar to tissue in vivo and the remaining 58% ( *n* = 7) considered the consistency were quite similar. A total of 80% of the novices absolutely yes, 75% of the intermediate group absolutely yes, and in the advanced group 33% of participants absolutely yes with this statement ( Graph 2 ). In question number 3, 42% ( *n* = 5) of the participants said very similar that the dissection of human placental vessels is similar to the dissection performed on vessels in vivo. As follows, in the novice group, 80% quite similar, 75% of the participants in the intermediate group very similar. And in the advanced group, each of the 3 experts evaluated this statement differently, as follows: very similar, similar and different ( Graph 3 ). Regarding questions numbers 4 all the participants answered absolutely yes that this simulation model could improve their surgical technique and reduce possible errors, and all of them yes that they would propose its use in microsurgical training. Question number 6 allows us to determine that 58% of the participants did not identify disadvantages to the use of this model, and the rest of the participants reported limited accessibility and preparation time for the model as drawbacks. Finally, question number 7 inquired about the limitations evidenced during the dissection and anastomosis, finding that 59% of the participants indicated some type of limitation during the dissection and/or anastomosis, such as: vessels very adherent to the underlying tissue, tunica media too thin, thick adventitia, and general difficulties during vessel dissection because the tissue is not identical compared with the dissection of septocutaneous or myocutaneous vessels ( Table 2 ).

**Table 2:** Results of the questionnaire to assess the reliability of the placenta as a microsurgical simulation model

No	Question ( *n* = 12) a	Results
1	Do you think that the proposed training model faithfully reproduces a possible real microsurgical scenario?	58% absolutely yes (CI: 27–84%) 42% somewhat (CI: 16–73%) 0% absolutely no
2	According to your surgical experience, is the consistency of the placental vessels comparable to the one of the in vivo tissues?	42% very similar (CI: 16–73%) 58% quite similar (CI: 27–84%) 0% different
3	Do you think that the dissection of human placenta vessels is similar to in vivo vessels dissection?	42% very similar (CI: 16–73%) 42% quite similar (CI: 16–73%) 16% different
4	Do you think that practice on this type of surgical training model can improve the surgical technique and reduce errors on the patient?	100% absolutely yes 0% somewhat 0% absolutely no
5	Do you think you will reuse or propose to use of this microsurgical training model?	100% yes 0% no
6	What disadvantages do you find in using this model?	58% none (CI: 27–84%) 25% model preparation time (CI: 7–61%) 17% limited accessibility (CI: 3–54%)
7	What inconveniences or difficulties did you encounter during the dissection and anastomosis of the placental vessels?	42% none (CI: 16–73%) 42%vessels firmly adherent to underlying tissue (CI: 16–73%) 16% medium thin tunic (CI: 17–100%) 16% thick adventitia (CI: 17–100%) 16% dissection is not similar to tissue in vivo (CI: 17–100%) General difficulties in dissection (8%; CI: 3–54%)

Abbreviation: CI, confidence interval.

a All the participants answered the questionnaire ( *n* = 12).

## Discussion

This study provides a simple, step-by-step, and reproducible method of using a dye-perfused training simulator with dynamic flow for plastic surgery residents and other specialties using microsurgery laboratories.

Currently, the rat is the standard model for microsurgical training due to its greater fidelity, the biomechanical qualities of the blood vessel in operating rooms as well as the possibility it offers of a better evaluation of anastomotic patency. 13 Although human placenta is a low-cost, available ex vivo simulation model that provides an alternative to the use of live animals; considering the abundant vasculature of the human placenta this model allows extended practice for the refinement of the microsurgical skills.

Since 1979, work has been done on the anatomical and histological identification of the placenta and its application as a training model. McGregor et al 14 examined 10 human placentas to describe the detailed histology of the wall vessel, which was characterized by a thick adventitia and a smaller caliber of tunica media. These findings were perceived in the same way by 59% of the participants in the study, who reported a smaller caliber of the tunica media, a perception of an increase in the thickness of the adventitia, and a marked adherence of the placental vessels to the underlying tissue.

The limitations of using placental tissue compared with the standard model that must be taken into account, such as the unfeasibility of performing a dissection by planes, the lack of neuroanatomical structures, the in vivo effects of coagulation, and the absence of pulsatile arterial blood flow 1 ; in this study it was found other disadvantages that are not mentioned in previous studies like the reduction in time up to 6 to 8 hours when working at 20 to 22°C of temperature, before the tissue turns friable and more delicate; therefore, if it is necessary to carry out more than one practice in the same day it will be mandatory to make new model preparations; furthermore, the use of placenta could have religious or ideological barriers (cultural) in different countries where people have beliefs about using placenta for experimentation. Finally, it is important to take into consideration that this is a multidisciplinary work that requires excellent communication between the gynecobstretic and plastic surgery departments in order to obtain fresh placentas for the preparation.

These disadvantages become more significant as the level of difficulty in the training exercises increases, but they do not interfere with the acquisition of basic microsurgical skills because all the basic techniques of microvascular surgery can be performed. They also permit the surgeon's initial familiarization process with the surgical loupes, microscope, the instruments, and ergonomics. Besides, to increase its similarity to a more realistic surgical scenario and evaluate the patency of anastomoses, our model uses a simple dynamic dye perfusion system.

Regarding anatomical findings, the study found the weight, surface diameter measurements of the placenta are comparable to previous literature. 1 6 7 15 16 In contrast, the average central thickness was smaller, and the most frequent location of the umbilical cord was eccentric, which differs from other descriptions in the literature. 1 The diameter of the blood vessels decreased as they approached the placental periphery also each segment or division, the average diameter of the veins was greater compared with the diameter of the arteries, findings that correlate with the preceding reports. 7 12 The upper and lower arterial diameter is similar to those reported in the literature. 7 8 12 14 Venous diameters, on the other hand, were smaller compared with those reported by previous authors. The importance of these anatomical analysis lies in extrapolate them to our population and apply them in microsurgery simulation laboratories. We found smaller venous vessels, which could offer a better environment for skill acquisition of atraumatic microsurgical technique using vessels of 0.8 to 1.5 mm. 17 ( Table 3 ).

**Table 3:** Comparison of reported placenta vessel diameters by author

Blood vessels	Reported average diameter (mm)					
	McGregor et al (1983) 14	Ayoubi et al (1992) 16	Fasano et al (1994) 8	Ramírez-Barba (1995) 19	Belykh et al (2016) 7	Ribeiro de Oliveira (2016) 18
Arteries	Hilum: 3.1 (1.0–5.0) Periphery: 0.95 (0.5–2.0)	1.0–6.0	Periferia: 0.5–1.5	2.1 (1.4–3.7)		
A1					6.5 ± 1.4 (3.0–9.0)	5.98 (3.73–8.29)
A2					3.4 ± 0.7 (2.0–5.0)	4.27 (2.54–7.89)
A3					1.7 ± 0.4 (0.8–3.0)	3.22 (1.28–5.80)
A4						2.62 (1.22–4.22)
Veins	Hilum: 3.9 (2.0–7.0) Periphery: 1.6 (0.5–3.0)					
V1					–	10.22 (9.40–12.27)
V2					–	7.40 (4.80–10.92)
V3					–	6.0 (4.60–8.21)
V4					–	4.20 (3.20–7.0)

Note: 1, first division; 2, second division; 3, third division; 4, fourth division.

As a background to the present study, the University of Pavia in Italy implemented a practical training course in vascular microneurosurgery, 1 where Del Maestro et al conducted a study with 42 dye-perfused placentas, in which 33 participants with different levels of experience in microsurgery performed various training exercises, including: end-to-end, end-to-side, and side-to-side dissection and anastomosis. At the end of the training course, the participants completed a five-question questionnaire to evaluate the validity and reliability of the color-perfused human placenta model for training in vascular microsurgery, and most of them, considered the human placenta as a valuable, accurate, and reproducible model.

In the present study the participants were manly young residents and the experience surgeons, most of them were men. The results shows similarly to the previous study, the participants generally reported agreeing that the consistency and dissection of human placental vessels are similar to the dissection performed on vessels in vivo and highlighted the ability of the model to perform a faithful reproduction of a real microsurgical scenario. In the statement about whether the dissection of the vessels is similar to the vessels in vivo, two out of three experts consider that the dissection is more demanding because it resembles tissue that has undergone radiation, which could be an interesting advantage because it will demand more meticulous and atraumatic dissection when faced more fibrous tissue, making the dissection of healthy tissues easier. In the analytic description, it was found that there was no statistical difference in answers of the participant regarding the subgroup of expertise, considering the results have a good fidelity. All of these results allow us to recognize the human placenta as an optimal tool to be considered in training within the microsurgery laboratory.

Compared with in vivo animal models that are subject to ethical and legal implications and also more expensive, requiring care in animal centers and the use of anesthetic techniques, 2 3 4 the human placenta demands less infrastructure, reducing costs and increasing availability at any hospital institution that has an obstetrics service close the microsurgery laboratory and the ethics committee permission. 18

The method presented allows a simple model of training in plastic surgery departments, resuming previous findings of using placental vascular trees, highlighting the validity of this model across different specialties, regardless of the experience of the trainees, and describing other disadvantages not previously well described to take into account when including this model into the options for microsurgery laboratories, such as the variability in time for using the model depending on the temperature, religious or ideological barriers, and an articulated communication within departments.
